# Reduction of left ventricular longitudinal global and segmental systolic functions in patients with hypertrophic cardiomyopathy: Study of two-dimensional tissue motion annular displacement

**DOI:** 10.3892/etm.2014.1617

**Published:** 2014-03-12

**Authors:** LIWEN LIU, SHENGJUN TUO, JIANLEI ZHANG, LEI ZUO, FANG LIU, LILI HAO, YANDAN SUN, LIPING YANG, HONG SHAO, WEI QI, XIAODONG ZHOU, SHUPING GE

**Affiliations:** 1 Department of Ultrasound, Xijing Hospital, The Fourth Military Medical University, Xi’an, Shaanxi 710032, P.R. China; 2 Department of Ultrasound, Yan’an People’s Hospital, Yan’an, Shaanxi 716000, P.R. China; 3 Department of Cardiology, The Heart Center, St. Christopher’s Hospital for Children, Drexel University College of Medicine, Philadelphia, PA 19134, USA

**Keywords:** echocardiography, hypertrophic cardiomyopathy, left ventricular function, two-dimensional strain, tissue motion annular displacement

## Abstract

The early detection of abnormal left ventricular systolic functions in patients with hypertrophic cardiomyopathy (HCM) remains a challenge. The aim of this study was to identify a novel method for the assessment of left ventricular systolic function in patients with HCM. A total of 65 patients with HCM were included in this study. The patients were divided into obstructive HCM (HOCM; 16 cases) and non-obstructive HCM (NOHCM; 49 cases) groups. The healthy control group comprised 48 participants. Two-dimensional (2D) speckle-tracking technology was used to measure the left ventricular global and segmental longitudinal strains and mitral annular displacement (MADs). Compared with healthy control group, the six segmental strains and the global strain of the left ventricle (LS_global_) increased while six segmental MADs and MAD_global_ of the mitral annulus decreased in the HOCM and NOHCM groups (P<0.05). In addition, the six segmental MADs of the mitral annulus were significantly negatively correlated with the six segmental strains of the left ventricle (r=−0.744 to −0.647, P<0.001). MAD_global_ was significantly negatively correlated with LS_global_ (r=−0.857, P<0.001). The tissue motion annular displacement (TMAD) at the midpoint was significantly negatively correlated with LS_global_ (r=−0.871, P<0.001). The 2D TMAD technique of measuring MAD was feasible and practically approachable for rapidly evaluating the left ventricular longitudinal global and segmental systolic functions of patients with HCM.

## Introduction

Modern molecular biological studies have confirmed that hypertrophic cardiomyopathy (HCM) is an autosomal dominant genetic disease caused by the genetic mutation of the myocardial sarcomere gene (1–4), and it has an incidence rate of ~1/500 (5,6). HCM is a common cause of sudden cardiac death in children and young individuals (7). The examination of the left ventricular functions is important for patients with HCM, particularly in the evaluation of treatment efficacy and long-term follow-up. The conventional view considers that cardiac involvement is common with HCM and represents as left ventricular diastolic dysfunction (8–11). However, molecular studies have identified that in HCM, the disorder of systolic function occurs initially and the cardiac hypertrophy is a compensatory response (12,13). Currently, sufficient evidence indicates that despite the reductions of left ventricular systolic functions in patients with HCM (14,15), such as significant reductions in the left ventricular strain and strain rate, the left ventricular ejection fraction (LVEF) of the majority of patients remains normal or enhanced (16). Therefore, the use of LVEF to evaluate the global left ventricular systolic functions is not able to truly reflect the impaired condition of the left ventricular systolic functions.

A number of novel ultrasound technologies, including tissue Doppler imaging (TDI), TDI-derived strain imaging and two-dimensional (2D) speckle tracking, are used to detect the reductions in left ventricular longitudinal systolic functions in patients with HCM. However, these techniques significantly depend on image quality and are time-consuming. The tissue motion annular displacement (TMAD) technique is a novel technology developed in accordance with speckle-tracking technology. This technique is able to assess the left ventricular longitudinal systolic functions by tracking the positions of the mitral annulus in the left ventricular systolic phase. TMAD quickly evaluates the left ventricular longitudinal systolic functions and does not require high image quality (17). In the present study, TMAD technology was used to assess the left ventricular global and segmental systolic functions in patients with HCM to provide a novel method of evaluating the left ventricular longitudinal systolic functions in these patients.

## Subjects and methods

### Subjects

A total of 65 patients with HCM were selected. The inclusion criteria (18) were as follows: 2D ultrasound showed asymmetric hypertrophy in the left ventricular walls; septum/posterior wall ratio was ≥1.5; the thickness of the interventricular septum was ≥15 mm; no significant or mild thickening was found in segments of LV wall except interventricular septum. The exclusion criteria were as follows: Ventricular hypertrophy caused by simple apical HCM; hypertension; coronary heart and valvular diseases; and LVEF <50%. According to the resting pressure gradient of the left ventricular outflow tract (PGlvot), the subjects were divided into the obstructive HCM (HOCM) group [PGlvot ≥30 mmHg] and the non-obstructive HCM (NOHCM) group (PGlvot <30 mmHg). The HOCM group comprised 16 cases (nine males and seven females, with a mean age of 42.7±11.7 years) and the NOHCM group comprised 49 cases (30 males and 19 females, with a mean age of 43.0±17.4 years). The 48 participants in the healthy control group were selected in the same period and according to the following conditions: Gender- and age-matched (30 males and 18 females, with a mean age of 41.2±14.5 years); normal results in the physical examinations, echocardiography, electrocardiography (ECG) and biochemical tests; and did not have coronary heart disease, hypertension, valvular disease, various arrhythmias and various systemic diseases. This study was conducted in accordance with the Declaration of Helsinki and with approval from the Ethics Committee of The Fourth Military Medical University (Xi’an, China). Written informed consent was obtained from all participants. The general information of the study subjects is shown in Table I.

### Standard ultrasound examination

The iE33 xMatrix UCG system (S5-1 transducer; Philips Healthcare, Andover, MA, USA) with external QLAB quantitative analysis software, version 8.1 (Philips Healthcare) was used. The left ventricular end-diastolic diameter, interventricular septum and posterior left ventricular wall thickness were measured along the left ventricular long-axis view. The septum/posterior wall ratio was calculated. The pulsed wave (PW) sampling volumes were placed under the mitral valves of the apical four-, two- and three-chamber views. The early and late mitral inflow velocities (E and A, respectively) were measured, and E/A was calculated. In the TDI mode, PW sampling volumes were placed in the interventricular septum, lateral wall, anterior wall, inferior wall, anterior septum, and the ring side of the posterior-wall mitral valve. Early and late myocardial diastolic velocities (Em and Am) were measured, and the mean values were obtained for the calculation of Em/Am and E/Em. When the PW sampling volume was placed on the aortic valve of the aortic long-axis view, the PGlvot was measured. The measurements of the aforementioned ultrasound data were based on the American Society of Echocardiography guidelines and requirements (19).

### Strain imaging data acquisition

**Figure 1:**
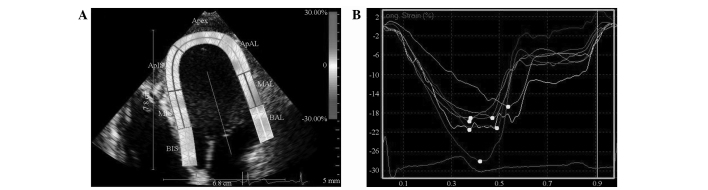
Apical four-chamber view STI measurement of left ventricular longitudinal strain and a strain-time curve. (A) Speckle-tracked overlay images of ROIs. The software was started and three points were selected. The software automatically traced the left ventricular wall, posterior septum and apical endocardium and epicardium, and equally divided the LV wall into the ventricular wall as basal, middle and apical segments. (B) Left ventricular lateral wall, posterior septum and apical strain-time curves. The average values of the basal, middle and apical segment of the corresponding ventricular wall were calculated to represent the strain values of the ventricular wall. STI, speckle-tracking imaging; ROI, region of interest.

All subjects were imaged in the left lateral position, with synchronous ECG display. The 2D images of three consecutive cardiac cycles of the apical four-, two- and three-chamber views were collected. The depth and fan size were adjusted to reach the maximum frame frequency (>50 frames/sec). Data were stored in a removable hard disk for offline analysis. QLAB software, version 8.1 was used to perform the following: Importation of the 2D grey-scale images; selection of the cardiac motion quantification (CMQ) module; sketching of the endocardium and epicardium in the region of interest (ROI) interface; and adjustment of the ROI width to match the myocardial thickness. After completely outlining the endocardium and epicardium, the software was run to automatically track the ROI cardiac echo spots and provide all myocardial segmental longitudinal strain curves and peaks (Fig. 1). The left ventricular longitudinal segmental strain was read. The average values of the basal, middle and apical segment of the left ventricular wall represented its strain. The ventricular wall segments selected for the analysis were the interventricular septum, lateral wall, inferior wall, anterior wall, anterior septum and posterior wall. The strains measured by the software included the basal, middle and apical segments of the corresponding wall, the mean value of which represented the longitudinally segmental strain (LS_seg_) of the ventricular wall. When more than two segments of the wall were not effectively tracked, the track was considered ineffective.

### TMAD data acquisition

**Figure 2:**
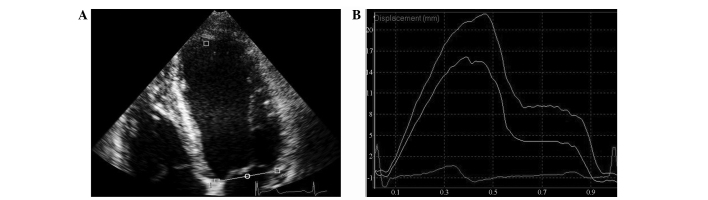
Apical four-chamber view for MAD measurement by the TMAD method and a displacement-time curve. (A) The software was started, three points were selected (lateral interventricular septum of mitral ring, lateral wall side of mitral ring and the apex), and the mitral annulus sampling points were marked. (B) Left ventricular lateral wall and posterior septum displacement-time curves. The displacement-time curves corresponded with the sampling points (in color, not shown), which enabled them to be quickly read. MAD, mitral annular displacement; TMAD, tissue motion annular displacement.

TMAD data were measured in the apical four-, two- and three-chamber views. The 2D grey-scale images were imported into QLAB software, version 8.1. Subsequently, the CMQ module was selected to outline three points in the ROI. Two points were placed in each of the interventricular septum and lateral wall side of the mitral ring of the apical four-chamber heart, the anterior interventricular septum, and the posterior wall side of the apical three-chamber heart, and anterior wall side and inferior wall side of the apical two-chamber heart. The third point was fixed on the surface of the apical endocardium (Fig. 2). The software automatically tracked the synchronous displacement curves of two sampling points and the corresponding segmental mitral annular values of displacement (MAD_seg_). The average value of the six wall segments was considered to represent the global displacement of the mitral ring (MAD_global_). The ratio of the midpoint MAD of the apical four-, two- and three-chamber views to the left ventricular long-axis was recorded as normalized midpoint displacement of the mitral ring [TMAD midpt (%)].

### Intra- and interobserver variability

Five healthy subjects and five patients with HCM were randomly selected, in which the TMAD of a total of 60 segments was measured. The MAD values of all segments were measured by doctors A and B. The difference reflected the interobserver error. Doctor B repeated the measurement of the same index of the above points two weeks later. From this measurement, the difference was used to reflect the intraobserver error. The degree of variation of these differences was then calculated. The error percentage of each measured value was used as the variability index, that is, (x1−x2)/[(x1+x2)/2] × 100, where ×1 is the result of the first measurement and ×2 is the result of the second measurement.

### Statistical analysis

All data were analysed using SPSS statistical analysis software, version 16.0 (SPSS, Inc., Chicago, IL, USA). The normally distributed measurement data were expressed as mean ± standard deviation. The quantitative parameters of the three groups were compared using analysis of variance. The least significant difference method was used for pairwise comparison. Pearson’s correlation was used to analyse the intergroup relationship and the χ^2^ test was used to compare the intergroup countable data. P<0.05 was considered to indicate a statistically significant difference.

## Results

### General information

No statistical differences were identified in the gender ratio, age, height, weight, body surface area, heart rate or blood pressure among the three groups (P>0.05; Table I).

### Conventional echocardiographic parameters

The interventricular septal thickness (IVST), left ventricular posterior wall thickness (LVPWT), septum/posterior wall ratio and left atrial volume (LAV) of the HOCM and NOHCM groups were increased compared with those of the healthy control group. Additionally, the left ventricular end-diastolic volume (LVEDV), early peak mitral inflow velocity (MV E), Em and peak myocardial systolic velocity (Sm) of the HOCM and NOHCM groups decreased, and the late peak mitral inflow velocity (MV A) and E/Em ratio of the HOCM and NOHCM groups increased compared with those of the healthy control group, whereas Am decreased. No significant differences were identified in the ejection fraction (EF), left ventricular end-systolic volume (LVESV) and left ventricular internal diameter at end diastole (LVIDd) among the three groups (Table II).

### Comparison of the left ventricular segmental strains and global strain (LS_global_)

The LS_seg_ and LS_global_ of the HOCM and NOHCM groups significantly increased compared with the healthy control group (P<0.01). No statistically significant difference was identified in the left ventricular segmental strains and LS_global_ between the HOCM and NOHCM groups (Table III).

### Comparison of segmental MADs and MAD_global_

The six MADs and MAD_global_ in the HOCM and NOHCM groups were significantly reduced compared with those of the healthy control group (P<0.01). No statistically significant differences were identified in the MAD_global_ and segmental MAD values of the HOCM and NOHCM groups (Table IV).

### Correlation analysis of the displacements of the six mitral ring sites and the left ventricular wall strain

**Figure 3:**
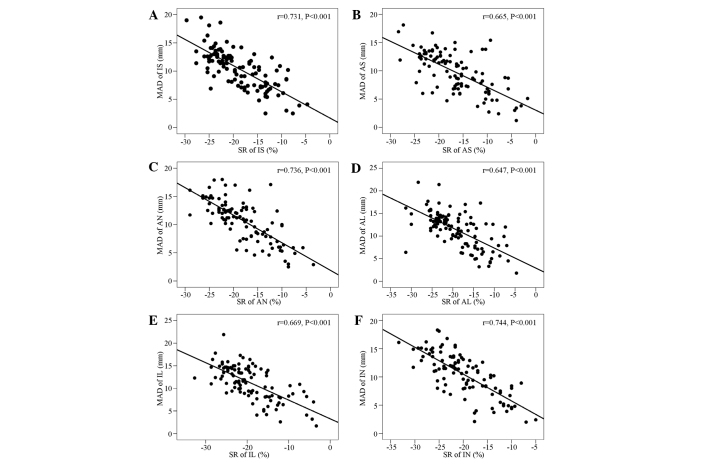
Correlation scatterplots of the displacements of six mitral ring sites with the left ventricular wall strain. MAD, mitral annular displacement; SR, strain; IS, inferoseptal; AS, anteroseptal; AN, anterior; AL, anterolateral; IL, inferolateral; IN, inferior.

Significant negative correlations were identified between the displacements of the six mitral ring sites and the left ventricular wall strain (Fig. 3).

### Correlation analysis of MAD_global_ and TMAD midpt with the LS_global_

**Figure 4:**
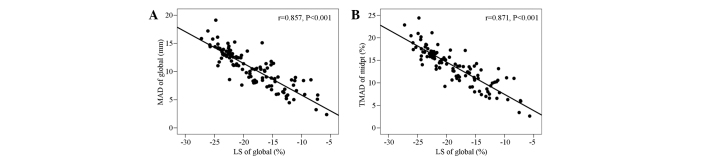
Correlation scatterplots of the mitral ring (A) global and (B) midpoint normalized displacement with the left ventricular global strain. MAD, mitral annular displacement; TMAD, tissue motion annular displacement; LS, left ventricular strain.

The MAD_global_ was significantly negatively correlated with the LS_global_ (r=−0.857, P<0.001). The TMAD midpt (%) was also significantly negatively correlated with LS_global_ (r=−0.871, P<0.001; Fig. 4).

### Repeatability test

No significant differences were identified in the TMAD and speckle-tracking imaging (STI) between the intra- (5.24±2.60 vs. 5.83±2.51) and interobserver variabilities (6.99±3.32 vs. 6.47±2.80) (P>0.05).

## Discussion

TMAD technology was used in the present study to evaluate the left ventricular longitudinal systolic functions of patients with HCM. Through a comparison conducted using 2D-STI, the LVEF in the HCM group was found to be non-significantly reduced compared with that in healthy controls. However, the left ventricular global and segmental longitudinal systolic functions of the patients with HCM were observed to be significantly reduced. In addition, the MADs were significantly negatively correlated with the left ventricular segmental strains. Furthermore, MAD_global_ and TMAD midpt values were significantly negatively correlated with the LS_global_ values. Therefore, we considered that it is possible to use TMAD to quickly and accurately assess the left ventricular longitudinal global and segmental functions of patients with HCM. Additionally, MAD may be more sensitive than LVEF in terms of identifying the left ventricular global and segmental systolic dysfunction.

The main pathological changes associated with HCM are myocardial hypertrophy, muscle cell disorder, contractile protein dysfunction and interstitial fibrosis (20). Of these changes, the first to occur is the left ventricular systolic dysfunction, whereas the myocardial hypertrophy is the compensatory response (12,13). More advanced technologies and further studies (14,15,21,22) have confirmed that the left ventricular longitudinal systolic functions in patients with HCM are reduced. Therefore, the detection of left ventricular longitudinal systolic functions in patients with HCM is important for the early detection of systolic dysfunction, disease progression monitoring, therapeutic review and prognosis evaluation. However, these novel techniques significantly depend on image quality, and assessment using poor quality images would be difficult to accomplish.

The mitral annulus is part of the fibrous skeleton of the heart. This structure has a large number of muscle fibres attached to the skeleton, which are arranged longitudinally throughout the space between the apex and base (23). The contraction of longitudinal muscle fibres shortens the ventricular cavity along the long-axis, leading to the mitral annulus moving downward towards the apex and the intraventricular blood gravity being relatively fixed in the position of the apex (24). Therefore, it is possible to use the apex as a reference point. It may be possible to use the displacement of the mitral annulus as it moves towards the apex to reflect left ventricular shortening. A study (25) detected that the shortening of the left ventricular long-axis contributed to almost 70% of the LVEF. Studies have demonstrated that the MAD may have important clinical value in the application of the left ventricular longitudinal functions to the evaluation of early diagnosis of heart damage (26), heart disease efficacy evaluation (27), prediction of cardiovascular events (28) and prognosis of certain heart diseases (29).

The present study observed that the MADs of the six mitral annulus points in the HCM group were lower compared with those of the healthy control group. This result suggests that the reduction of the left ventricular segmental systolic functions in the patients with HCM not only occurs in the hypertrophic myocardial segments, but also in the non-hypertrophic myocardial segments. The left ventricular longitudinal myocardial fibres are mainly distributed under the endocardium, which may be more susceptible to pathological factors. The MAD is the indirect reflection of the contractile function of these longitudinal myocardial fibres. Therefore, the evaluation of the MAD on the left ventricular longitudinal systolic function has potential value in the early diagnosis of the left ventricular damage associated with HCM. Certain studies of patients with diastolic heart failure have shown that isolated diastolic heart failure is rare. The majority of cases are associated with left ventricular longitudinal systolic dysfunction. Nishikage *et al* (26) observed that in patients with hypertension with a normal LVEF, the left ventricular systolic dysfunction occurred prior to the longitudinal diastolic dysfunction. Thus, MAD is an early reflection of the left ventricular systolic dysfunction. In addition, MAD is able to sensitively reveal the left ventricular systolic dysfunction. As indicated by the present study, the detection and evaluation of the left ventricular early systolic dysfunction associated with HCM by measuring the MAD is likely to provide a novel perspective.

The present study revealed that the LVEF did not significantly differ between the HCM and control groups, regardless of the type of HCM (cHCM or nHCM). However, the representative index of left ventricular global systolic function, namely, MAD_global_, was reduced, which suggested that patients with HCM suffered from left ventricular global longitudinal systolic dysfunction. However, MAD_seg_ and MAD_global_ were the actual displacements, by which the mitral annulus moved directly to the apex. These displacements were not considered in relation to the actual heart size. Therefore, comparisons among different individuals were adversely affected. Similarly to the LVEF, the ratio of TMAD midpt to the left ventricular long axis was used. It was observed that the left ventricular TMAD midpt of the patients with HCM was reduced. The contraction of the heart and volume deformation includes the shortening of the cardiac long and short axes. The TMAD midpt may be a practical and feasible indicator for evaluating the longitudinal shortening rate in patients with HCM, which is worthy of further study.

The 2D-STI method is able to track 2D ultrasound myocardial spot positions through a frame-by-frame process and determine the myocardial strain through the spot position-change rate to reflect the left ventricular contractile function (14); it overcomes angle dependence and possesses a good correlation with cardiac magnetic resonance (30,31). 2D-STI also accurately assesses the left ventricular global and segmental systolic functions. In the present study, the detection of left ventricular 2D strain in HCM through 2D-STI and further comparison with the TMAD data revealed that the 2D-STI detection of LS_global_ and the left ventricular segmental strains had a good correlation with the TMAD detection of the MAD_global_ and MAD_seg_. Therefore, TMAD was able to accurately assess the left ventricular longitudinal global and segmental systolic functions. TMAD is advantageous due to its low-quality image requirements and rapid measurement (17).

In conclusion, TMAD was able to accurately and quickly evaluate the left ventricular longitudinal global and segmental systolic functions with low-quality image requirements and good reproducibility. In addition, TMAD may be used for the evaluation of left ventricular longitudinal systolic functions in patients with HCM. The proposed technique exhibits possible potential prognostic value in the early diagnosis, disease progression monitoring, efficacy assessment and prognosis evaluation of HCM.

This study included a small number of cases. Therefore, it was not possible to determine the MAD normal reference values. Furthermore, the impact factors affecting MAD remain unclear. MAD was simply the reflection of the ventricular wall segmental overall function at the mitral annular attachment point, and was not able to reveal the systolic functions of the corresponding basal, middle and apical segments. Therefore, the information regarding left ventricular motion in the patients with HCM provided by the TMAD data was less than that provided by the strain data. All limitations of this study necessitate further study.

## Figures and Tables

**Table I:** Clinical characteristics of the three groups.

Parameter	HOCM (n=16)	NOHCM (n=49)	Controls (n=48)	P-value
Male, n (%)	9 (56.3)	30 (61.2)	30 (62.5)	0.906
Age (years)	42.7±11.7	43.0±17.4	41.2±14.5	0.836
Height (cm)	165.3±7.0	167.4±8.1	167.7±6.0	0.601
Weight (kg)	64.6±10.8	67.3±12.8	63.9±9.6	0.314
BSA (m^2^)	1.7±0.2	1.8±1.1	1.7±0.1	0.649
HR (beats/min)	71.0±12.2	74.0±11.9	71.8±9.8	0.535
Systolic BP (mmHg)	113.6±10.4	110.2±10.5	109.5±10.7	0.451
Diastolic BP (mmHg)	67.5±6.9	68.8±6.4	68.0±4.8	0.700
Drug therapy, n (%)				
ACE inhibitor	8 (50.0)	17 (34.7)	0	-
ARB	4 (25.0)	21 (42.9)	0	-
β-blocker	7 (43.8)	16 (32.7)	0	-
Calcium-channel blocker	7 (43.8)	18 (36.7)	0	-
Aspirin	10 (62.5)	31 (63.3)	0	-

HOCM, obstructive hypertrophic cardiomyopathy; NOHCM, non-obstructive HCM; BSA, body surface area; HR, heart rate; BP, blood pressure; ACE, angiotensin-converting enzyme; ARB, angiotensin II receptor blocker.

**Table II:** Echocardiographic characteristics of the three groups.

Parameter	HOCM	NOHCM	Controls	P-value
IVST(mm)	24.3±3.1^a^	20.2±3.5^a^	7.5±1.1	<0.001
LVPWT (mm)	11.5±3.2^a^	10.5±2.9^a^	7.1±1.1	<0.001
Septum/posterior wall ratio	2.2±0.6^a^	2.1±0.6^a^	1.1±0.1	<0.001
LVIDd (mm)	41.8±3.5	44.6±5.1	44.3±3.0	0.053
LVEDV (ml)	60.5±15.9^a^	67.0±17.6^a^	74.7±14.7	0.006
LVESV (ml)	23.3±7.0	27.0±9.2	27.6±5.3	0.134
EF biplane (%)	64.1±6.2	61.3±7.8	62.0±3.6	0.304
LAV biplane (ml)	48.7±18.9^a^	42.5±19.1^a^	27.1±6.2	<0.001
PGlvot (mmHg)	67.4±16.9^a^	10.7±7.8^a^	4.6±1.4	<0.001
MV E (cm/sec)	71.1±20.4^a^	71.0±22.0^a^	84.6±17.9	0.003
MV A (cm/sec)	92.0±30.2^a^,^b^	71.9±27.2^a^	63.7±15.9	<0.001
MV E/A	0.86±0.37^a^	1.11±0.54^a^	1.44±0.63	0.001
Sm (cm/sec)	5.9±2.1^a^,^b^	6.7±1.8^a^	8.3±1.6	0.032
Em (cm/sec)	3.5±1.0^a^	4.7±2.2^a^	9.3±2.2	<0.001
Am (cm/sec)	6.6±2.2^a^	6.5±1.8^a^	7.5±1.5	0.022
Em/Am	0.55±0.11^a^,^b^	0.76±0.39^a^	1.3±0.48	<0.001
E/Em	21.0±7.3^a^,^b^	16.8±7.0^a^	9.3±1.6	<0.001

a P<0.05, compared with controls;

b P<0.05, ccompared with nHCM.

cHCM, obstructive hypertrophic cardiomyopathy; nHCM, non-obstructive HCM; IVST, interventricular septal thickness; LVPWT, left ventricular posterior wall thickness; LVIDd, left ventricular internal diameter at end diastole; LVEDV, left ventricular end-diastolic volume; LVESV, left ventricular end-systolic volume; EF, ejection fraction; LAV, left atrial volume; PGlvot, pressure gradient of the left ventricular outflow tract; MV E, early mitral inflow velocity; MV A, late mitral inflow velocity; Sm, peak myocardial systolic velocity; Em, early myocardial diastolic velocity; Am, late myocardial diastolic velocity; E, early ventricular filling velocity.

**Table III:** Comparison of the left ventricular segmental and global strains of the three groups.

Parameter	HOCM	NOHCM	Controls	P-value
SR_IS_	−14.6±4.4^a^	−15.7±4.8^a^	−23.1±2.4	<0.001
SR_AL_	−14.9±4.1^a^	−16.2±5.5^a^	−23.1±3.0	<0.001
SR_AN_	−13.7±4.0^a^	−14.6±4.4^a^	−22.6±2.5	<0.001
SR_IN_	−17.0±4.8^a^	−17.3±6.1^a^	−23.8±3.5	<0.001
SR_AS_	−14.0±4.1^a^	−13.0±6.1^a^	−20.5±3.1	<0.001
SR_IL_	−17.0±5.3^a^	−16.4±6.8^a^	−19.3±6.1	<0.001
LS_global_	−15.2±3.2^a^	−15.6±4.5^a^	−18.6±5.0	<0.001

a P<0.001, compared with controls.

HOCM, obstructive hypertrophic cardiomyopathy; NOHCM, non-obstructive HCM; SR, strain; IS, inferoseptal; AL, anterolateral; AN, anterior; IN, inferior; AS, anteroseptal; IL, inferolateral; LS, left ventricular strain.

**Table IV:** MADs of the three groups.

Parameter	HOCM	NOHCM	Controls	P-value
MAD_IS_ (mm)	8.3±2.2^a^	8.4±2.9^a^	13.1±2.3	<0.001
MAD_AL_ (mm)	9.7±3.3^a^	9.3±3.8^a^	13.9±2.5	<0.001
MAD_AN_ (mm)	8.4±3.0^a^	9.1±3.7^a^	13.1±2.2	<0.001
MAD_IN_ (mm)	8.4±3.0^a^	8.2±3.2^a^	13.5±2.2	<0.001
MAD_AS_ (mm)	8.2±2.2^a^	7.5±3.3^a^	12.4±2.1	<0.001
MAD_IL_ (mm)	10.3±2.7^a^	8.8±3.6^a^	13.8±2.3	<0.001
MAD_global_ (mm)	8.9±1.9^a^	8.6±2.8^a^	13.3±1.8	<0.001
TMAD midpt (%)	11.5±2.4^a^	11.0±3.8^a^	16.7±2.4	<0.001

a P<0.001, compared with controls.

MAD, mitral annular displacement; HOCM, obstructive hypertrophic cardiomyopathy; NOHCM, non-obstructive HCM; IS, inferoseptal; AL, anterolateral; AN, anterior; IN, inferior; AS, anteroseptal; IL, inferolateral; TMAD, tissue motion annular displacement; midpt, midpoint.

## References

[1] Marian AJ, Salek L, Lutucuta S (2001). Molecular genetics and pathogenesis of hypertrophic cardiomyopathy. Minerva Med.

[2] Niimura H, Patton KK, McKenna WJ (2002). Sarcomere protein gene mutations in hypertrophic cardiomyopathy of the elderly. Circulation.

[3] Watkins H, Conner D, Thierfelder L (1995). Mutations in the cardiac myosin binding protein-C gene on chromosome 11 cause familial hypertrophic cardiomyopathy. Nat Genet.

[4] Waldmüller S, Sakthivel S, Saadi AV (2003). Novel deletions in MYH7 and MYBPC3 identified in Indian families with familial hypertrophic cardiomyopathy. J Mol Cell Cardiol.

[5] Fananapazir L, Epstein ND (1995). Prevalence of hypertrophic cardiomyopathy and limitations of screening methods. Circulation.

[6] Maron BJ, Gardin JM, Flack JM, Gidding SS, Kurosaki TT, Bild DE (1995). Prevalence of hypertrophic cardiomyopathy in a general population of young adults. Echocardiographic analysis of 4111 subjects in the CARDIA Study Coronary Artery Risk Development in (Young) Adults. Circulation.

[7] Maron BJ, Doerer JJ, Haas TS, Tierney DM, Mueller FO (2009). Sudden deaths in young competitive athletes: analysis of 1866 deaths in the United States, 1980–2006. Circulation.

[8] Maron BJ, Spirito P, Green KJ, Wesley YE, Bonow RO, Arce J (1987). Noninvasive assessment of left ventricular diastolic function by pulsed Doppler echocardiography in patients with hypertrophic cardiomyopathy. J Am Coll Cardiol.

[9] Chen YT, Chang KC, Hu WS, Wang SJ, Chiang BN (1987). Left ventricular diastolic function in hypertrophic cardiomyopathy: assessment by radionuclide angiography. Int J Cardiol.

[10] Nihoyannopoulos P, Karatasakis G, Frenneaux M, McKenna WJ, Oakley CM (1992). Diastolic function in hypertrophic cardiomyopathy: relation to exercise capacity. J Am Coll Cardiol.

[11] Matsumura Y, Elliott PM, Virdee MS, Sorajja P, Doi Y, McKenna WJ (2002). Left ventricular diastolic function assessed using Doppler tissue imaging in patients with hypertrophic cardiomyopathy: relation to symptoms and exercise capacity. Heart.

[12] Rust EM, Albayya FP, Metzger JM (1999). Identification of a contractile deficit in adult cardiac myocytes expressing hypertrophic cardiomyopathy-associated mutant troponin T proteins. J Clin Invest.

[13] Bottinelli R, Coviello DA, Redwood CS (1998). A mutant tropomyosin that causes Hypenrophic cardiomyopathy is expressed in vivo and associated with an increased calcium sensitivity. Circ Res.

[14] Serri K, Reant P, Lafitte M (2006). Global and regional myocardial function quantification by two-dimensional strain: application in hypertrophic cardiomyopathy. J Am Coll Cardiol.

[15] Abozguia K, Nallur-Shivu G, Phan TT (2010). Left ventricular strain and untwist in hypertrophic cardiomyopathy: relation to exercise capacity. Am Heart J.

[16] Wigle ED, Rakowski H, Kimball BP, Williams WG (1995). Hypertrophic cardiomyopathy. Clinical spectrum and treatment. Circulation.

[17] Buss SJ, Mereles D, Emami M (2012). Rapid assessment of longitudinal systolic left ventricular function using speckle tracking of the mitral annulus. Clin Res Cardiol.

[18] Gersh BJ, Maron BJ, Bonow RO (2011). 2011 ACCF/AHA guideline for the diagnosis and treatment of hypertrophic cardiomyopathy: a report of the American College of Cardiology Foundation/American Heart Association task force on practice guidelines. Developed in collaboration with the American Association for Thoracic Surgery, American Society of Echocardiography, American Society of Nuclear Cardiology, Heart Failure Society of America, Heart Rhythm Society, Society for Cardiovascular Angiography and Interventions, and Society of Thoracic Surgeons. J Am Coll Cardiol.

[19] Lang RM, Bierig M, Devereux RB, Chamber Quantification Writing Group; American Society of Echocardiography’s Guidelines and Standards Committee; European Association of Echocardiography (2005). Recommendations for chamber quantification: a report from the American Society of Echocardiography’s Guidelines and Standards Committee and the Chamber Quantification Writing Group, developed in conjunction with the European Association of Echocardiography, a branch of the European Society of Cardiology. J Am Soc Echocardiogr.

[20] Maron BJ (2002). Hypertrophic cardiomyopathy: a systematic review. JAMA.

[21] Weidemann F, Mertens L, Gewillig M, Sutherland GR (2001). Quantitation of localized abnormal deformation in asymmetric nonobstructive hypertrophic cardiomyopathy: a velocity, strain rate, and strain Doppler myocardial imaging study. Pediatr Cardiol.

[22] Yang H, Sun JP, Lever HM (2003). Use of strain imaging in detecting segmental dysfunction in patients with hypertrophic cardiomyopathy. J Am Soc Echocardiogr.

[23] Greenbaum RA, Ho SY, Gibson DG, Becker AE, Anderson RH (1981). Left ventricular fibre architecture in man. Br Heart J.

[24] Rodriguez F, Tibayan FA, Glasson JR (2004). Fixed-apex mitral annular descent correlates better with left ventricular systolic function than does free-apex left ventricular long-axis shortening. J Am Soc Echocardiogr.

[25] Carlsson M, Ugander M, Mosén H, Buhre T, Arheden H (2007). Atrioventricular plane displacement is the major contributor to left ventricular pumping in healthy adults, athletes, and patients with dilated cardiomyopathy. Am J Physiol Heart Circ Physiol.

[26] Nishikage T, Nakai H, Lang RM, Takeuchi M (2008). Subclinical left ventricular longitudinal systolic dysfunction in hypertention with no evidence of heart failure. Circ J.

[27] Zhang Q, Fung JW, Yip GW (2008). Improvement of left ventricular myocardial short-axis, but not long-axis function or torsion after cardiac resynchronisation therapy: an assessment by two-dimensional speckle tracking. Heart.

[28] Ballo P, Barone D, Bocelli A, Motto A, Mondillo S (2008). Left ventricular longitudinal systolic dysfunction is an independent marker of cardiovascular risk in patients with hypertension. Am J Hypertens.

[29] Sveälv BG, Olofsson EL, Andersson B (2008). Ventricular long-axis function is of major importance for long-term survival in patients with heart failure. Heart.

[30] Amundsen BH, Helle-Valle T, Edvardsen T (2006). Noninvasive myocardial strain measurement by speckle tracking echocardiography: validation against sonomicrometry and tagged magnetic resonance imaging. J Am Coll Cardiol.

[31] Korinek J, Wang J, Sengupta PP (2005). Two-dimensional strain - a Doppler-independent ultrasound method for quantitation of regional deformation: validation in vitro and in vivo. J Am Soc Echocardiogr.

